# Motion influences gaze direction discrimination and disambiguates contradictory luminance cues

**DOI:** 10.3758/s13423-015-0971-8

**Published:** 2015-11-12

**Authors:** Nicola C. Anderson, Evan F. Risko, Alan Kingstone

**Affiliations:** Department of Cognitive Psychology, Vrije Universiteit Amsterdam, Van der Boechorststraat 1, 1081 BT Amsterdam, The Netherlands; Department of Psychology, University of Waterloo, Waterloo, Ontario Canada; Department of Psychology, University of British Columbia, Vancouver, British Columbia Canada

**Keywords:** Social cognition, Visual perception, Gaze perception, Gaze discrimination

## Abstract

In two experiments, we investigated the role of apparent motion in discriminating left/right gaze deviation judgments. We demonstrated that discrimination accuracy and response confidence was significantly higher when the eyes were moved to the left or right, compared to when the eyes were presented in their final shifted position (static images). To dissociate the role of motion signals from luminance signals, gaze stimuli were also presented in reverse contrast. Replicating past studies polarity reversal had a profound and detrimental effect on gaze discrimination in static images, although, intriguingly, while response confidence remained low, participant performance improved as gaze angle increased. In striking contrast to these data, polarity reversal had no negative effect on performance when the eyes were moved. We discuss these findings in the context of a multiple-cue account of gaze perception.

The eyes represent an important social stimulus. They indicate when, and where, others might be directing their attention in the world, which helps to support complex social behaviour and cooperation among individuals (Maurer, 1985; Baron-Cohen, 1995; Kendon, 1967; Richardson & Dale, 2005; Foulsham, Cheng, Tracy, Henrich, & Kingstone, 2010; Gibson & Pick, 1963). To support these functions, the human attentional system has evolved to prioritize gaze as a critical stimulus (Risko, Laidlaw, Freeth, Foulsham, & Kingstone, 2012) and to react to gaze direction efficiently (even automatically; Laidlaw, Risko, & Kingstone, 2012). Indeed, there is much evidence suggesting that humans are experts at determining the location of another’s gaze (see Anderson, Risko, & Kingstone, 2011 for a review). In the present investigation, we focus on understanding the factors that support this ability.

Previous research has focused on two information sources contributing to the processing of gaze direction: luminance distribution and geometrical cues (Ando, 2004; Olk, Symons, & Kingstone, 2008). Luminance distribution across the eye is the relative amount of light and dark low-spatial frequency information from the sclera and iris that is visible to an observer. When the luminance distribution is altered, the discriminability of gaze direction becomes impaired or reversed (Sinha, 2000; Ricciardelli, Baylis, & Driver, 2000). Geometrical cues refer to the high spatial frequency information present in the form of the iris and its location within the sclera and surrounding eyelid. As such, the extent to which the circular (and darker) iris deviates relative to the amount of sclera visible between the eyelids signals the direction of gaze (Anstis, Mayhew, & Morley, 1969).

Both the luminance distribution and geometrical cues can be derived from a static image of an individual’s eyes. However, one’s eyes are rarely still for long periods of time. In the present investigation, we set out to determine whether this movement, in and of itself, also could be used as a cue when processing gaze. Motion is a rich signal that can provide rapid information about object speed, velocity, direction, and depth (Hubel & Wiesel, 1979; Maunsell & Van Essen, 1983; Movshon & Newsome, 1992; Nakayama & Silverman, 1985). Indeed, motion is said to be a “primary sensation” (Coren, Ward, & Enns, 1999, p. 405). Thus, it seems plausible that motion would provide a cue to the direction of gaze.

Interestingly, research to date assessing the contribution of eye motion to gaze discrimination has found little support for the idea that motion contributes to the perception of gaze direction. For example, in a gaze discrimination task, Symons, Lee, Cedrone, and Nishimura (2004) asked observers to indicate whether a model was looking to the left or right of a series of defined targets on a peg board, each separated by less than 0.5° of visual angle. The model either made eye contact with an observer, and then fixated a peg on the board, or occluded her eyes while they were moving. No difference in accuracy was found between these dynamic and static conditions. Similarly, Bock, Dicke, and Thier (2008) asked observers to triangulate a model’s gaze to any one of 90 pinheads around a circular board and again found no differences in precision between static and dynamic gaze.

The lack of a contribution of motion to gaze discrimination in previous work is surprising given the visual system’s sensitivity to motion (Coren et al., 1999). In the Symons et al. study, the model in the dynamic condition tended to overshoot the target position, which would have made it ineffective as a reliable information source, a possibility raised both by Symons and the participants in his study. However, this does not seem to be an issue in the Bock et al. study. An alternative explanation is that because the Symons and Bock studies presented many small closely packed targets to the observers, the task demanded precise gaze and target position information. In this case, the stationary luminance and geometric cues may be more informative than the motion cue. In other words, the gaze cue information after motion was complete was more reliable than the motion information. This would render the motion cue irrelevant and lead to the equivalent dynamic and static findings.

To explore these ideas across two experiments, we presented participants with gaze discrimination tasks that required (a) little or no specific localization of gaze direction beyond a broad spatial code consisting of whether gaze has been shifted to the left or the right (Experiment 1A), or (b) a relatively more fine grained discrimination between eye positions on a given side of space (Experiment 1B; e.g., did gaze shift 1° or 3° to the left or right). In all cases, special care was taken to keep the possible decisions to a minimum (e.g., left vs. right in 1A) and the visual angle relatively large compared to previous studies (e.g., 2° eye movement in 1B). In doing so, we sought to increase the likelihood of finding a contribution of motion to gaze discrimination, whereby observers are more accurate at discriminating dynamic than static gaze.

Explicit in the logic outlined above is the notion that gaze discrimination can draw on multiple cues and that the contribution of any single cue is likely dependent on the nature of the gaze discrimination required. The potential use of multiple cues in the discrimination of gaze direction also raises an important theoretical issue regarding how the different cues are integrated and interact. To investigate this question with respect to motion, we combined the manipulation of eye motion with changes in luminance distribution, one of the cues previously demonstrated to influence gaze discrimination. We use a reverse contrast manipulation, which is known to disrupt gaze perception for static images by inverting the typical relationship between a dark iris and a bright sclera by making the iris bright and the sclera dark (Olk et al., 2008; Ricciardelli et al., 2000; Sinha, 2000). If motion and luminance distribution cues contribute to gaze discrimination in a noninteractive fashion, then we would expect the effects of motion and contrast reversal to be additive. In other words, the “cost” of contrast reversal should be of equivalent size whether the eyes are moving or not. Alternatively, if motion and luminance distribution cues do interact, then the pattern of this interaction may help to elucidate their relative contributions in gaze perception. For example, the “cost” of contrast reversal might be ameliorated when a motion cue is present relative to when it is absent. In addition to allowing us to assess potential interactions between different cues, the inclusion of a contrast reversal manipulation also permits an assessment of the relative contribution of luminance cues to tasks requiring simple left vs. right discriminations (Experiment 1A) versus a discrimination within the same side of space (Experiment 1B).

Finally, we included a measure of response confidence to assess the relative importance of our manipulations on individuals’ subjective perception of the gaze signal (Cheesman & Merikle, 1986; but see Kunimoto, Miller, & Pashler, 2001). If motion is a particularly important cue for determining the direction of gaze, then we would expect confidence to be high in dynamic trials. If luminance is a particularly strong cue, then we might expect confidence to be higher for normal than reverse-contrast gaze.

## Method

### Participants

Twenty-four participants (*M*_age_ = 21.3, 18 females) from the University of British Columbia took part for course credit or 5 dollars (Experiment 1A) and 12 participants (*M*_age_ = 27.3, 9 males) from the VU University Amsterdam took part for 3 Euro (Experiment 1B).

### Apparatus

Stimuli were presented on a Dell 2407WFP 17-inch flat screen monitor (Experiment 1A) or Samsung Syncmaster 2233RZ 22-inch flat screen (Experiment 1B), and participants were seated approximately 60-80 centimetres away. Responses were recorded on a standard keyboard number pad.

### Stimuli

**Fig. 1:**
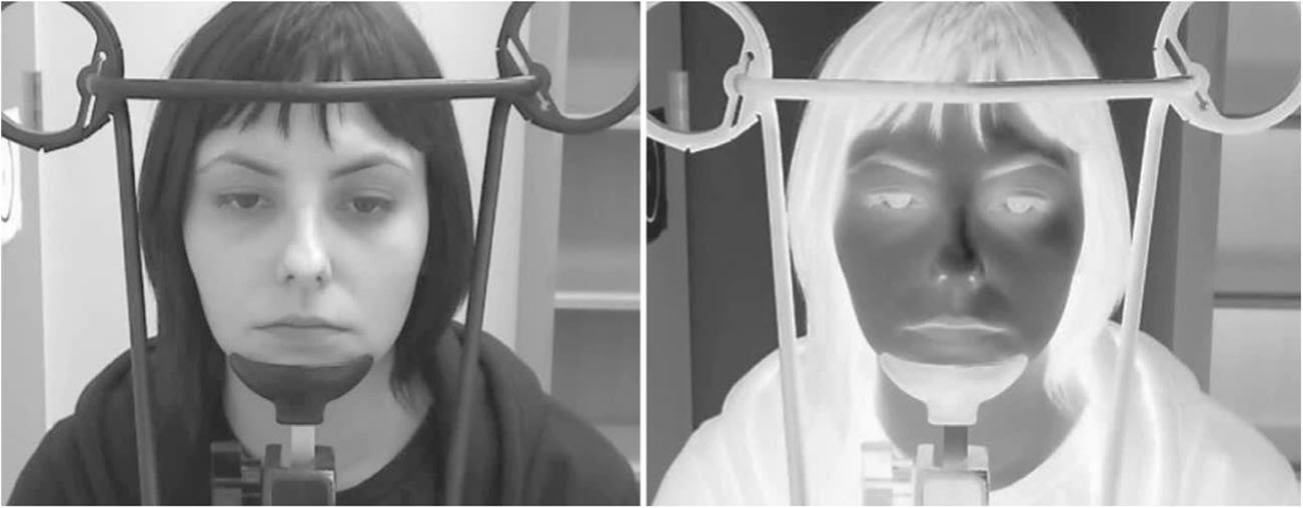
Example normal and reverse contrast stimulus. The model is looking 3 degrees to the left in both images

Stimuli were adapted from those developed in Anderson et al. (2011). In that study, videos depicted eye movements from two male and two female models. Eye movements were made from a central fixation point to targets on the left or right at 1°, 2°, or 3° (Experiment 1A) or 1° and 3° (Experiment 1B) away from the screen center. Each video lasted approximately 1.5 seconds. Each of the models contributed 8 left and 8 right eye movements for each gaze angle, for a total of 192 trials (Experiment 1A) and 128 trials (Experiment 1B). Two frames were then extracted from these videos. The first frame depicted the model looking at a central target on the screen in front of them (looking straight ahead). The second frame was extracted from the end point where the model is looking 1°-3° to the left or right. These frames were converted to greyscale and reverse contrast polarity (Fig. 1). To create the dynamic condition, the perception of smooth motion was produced by displaying the two frames (the first for 1000 ms) in succession. We chose apparent motion for two reasons. The first is that with the frame-rate of the original video (30 fps), it is unlikely that any frames would capture the intermediate motion of the eyes during a saccade. Second, presenting two frames rather than a video more closely equated the dynamic and static conditions. Specifically, in the static condition and consistent with standard methodology, only a single image—the second extracted image (the model looking left or right)—was presented.^1^

### Procedure and design

Participants were seated comfortably in front of the computer monitor and given detailed instructions about the procedure. The conditions were presented to participants in a random blocked design of 24 trials in each block: 4 blocks for normal contrast (2 static, 2 dynamic) and 4 reverse contrast blocks (2 static, 2 dynamic). Participants were told before each block whether the model will “have already looked to the left or right” (static trials) or “will look straight ahead, then to the left or right” (dynamic trials). All gaze stimuli were displayed in this fashion and were counterbalanced across participants. Thus, across four participants all eye movements would be seen in static, dynamic, normal, and reverse contrast form.

During each trial, participants were presented with the static or dynamic gaze stimulus (which was either normal or reversed in contrast, depending on the block). In Experiment 1A, participants were asked to respond to the direction that they perceived the eyes looked (dynamic) or was looking (static). Participants were required to respond with the number pad (arranged like a calculator) whether they perceived rightward gaze (press 6) or leftward gaze (press 4). In Experiment 1B, participants were asked to respond whether the eyes looked (dynamic) or were looking (static) 3° left or right (press 4 or 6, respectively) or 1° left or right (press 1 or 3, respectively). Stimuli remained on screen until response selection. After each response, participants were asked to rate their confidence in their decision. Confidence responses were given using the number pad where 0 corresponded to a “guess” response and 9 to an “absolutely sure” response.

## Results – Experiment 1A

### Accuracy

**Fig. 2:**
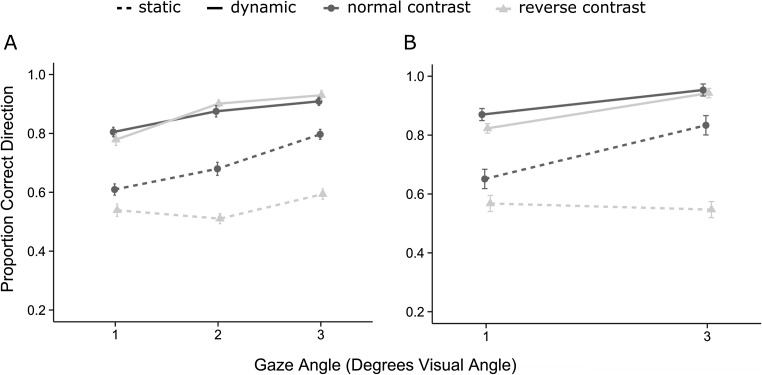
Panel **a** (Experiment 1A) and Panel **b** (Experiment 1B) depict the proportion of correct direction judgments for normal and reverse contrast gaze in the static and dynamic conditions for each gaze angle. Error bars in this and all remaining figures represent standard error corrected for between-subjects variance (Cousineau, 2005; Morey, 2008)

Figure 2a shows mean accuracy for normal and reverse contrast, static, and dynamic stimuli across the 3 gaze angles. A 2 (contrast: reverse contrast, normal contrast) × 2 (motion: static, dynamic) × 3 (gaze angle) within-subjects analysis of variance was conducted on the proportion of correct responses. Gaze angle was treated as a linear factor for this and all other measures and all reports relating to this factor represent the linear contrast. There was a main effect of contrast, *F*(1,23) = 16.43, *MSE* = 0.02, *p* = 0.001, *η*_*p*_^*2*^ = 0.417, such that accuracy was significantly better for normal contrast (*M *= 0.78) than reverse contrast stimuli (*M* = 0.71). In addition, there was a main effect of motion, *F*(1,23) = 107.87, *MSE* = 0.04, *p* < 0.001, *η*_*p*_^*2*^ = 0.824, such that accuracy was greater for dynamic (*M* = 0.87) compared with static stimuli (*M* = 0.62). There also was a main effect of gaze angle, *F*(1,23) = 67.12, *MSE* = 0.003, *p* < 0.001, *η*_*p*_^*2*^ = 0.745, such that accuracy increased as gaze angle increased.

Critically, there was a significant contrast by motion interaction, *F*(1,23) = 24.81, *MSE* = 0.07, *p* < 0.001, *η*_*p*_^*2*^ = 0.519, such that the effect of the reverse contrast manipulation was larger in the static condition *(M*_*diff*_ = 0.14) than in the dynamic condition (*M*_*diff*_ = −0.01), *t*(23) = 4.98, *p* < 0.001, *d* = 1.0. Indeed, there was no difference between normal and reverse contrast stimuli in the dynamic condition, *t*(23) = 0.25, *p* = 0.802, *d* = 0.02, whereas this difference was significant in the static condition, *t*(23) = 8.12, *p* < 0.001, *d =* 1.49.

The contrast by motion interaction was qualified by a significant contrast by motion by gaze angle interaction, *F*(1,23) = 13.01, *MSE* = 0.03, *p* = 0.001, *η*_*p*_^*2*^ = 0.361. For static stimuli, the effect of gaze angle on accuracy was significantly greater for normal contrast (0.10/degree) compared with reverse contrast (0.03/degree), *t*(23) = 3.37, *p* = 0.003, *d* = 0.75. For dynamic stimuli, no significant difference between the effect of gaze angle was found between normal (0.05/degree) and reverse contrast (0.08/degree) stimuli, *t*(23) = 1.42, *p* = 0.169, *d* = 0.32. Thus, the influence of contrast reversal increased with gaze angle for static stimuli but not for dynamic stimuli (Fig. 2a).

### Confidence

**Fig. 3:**
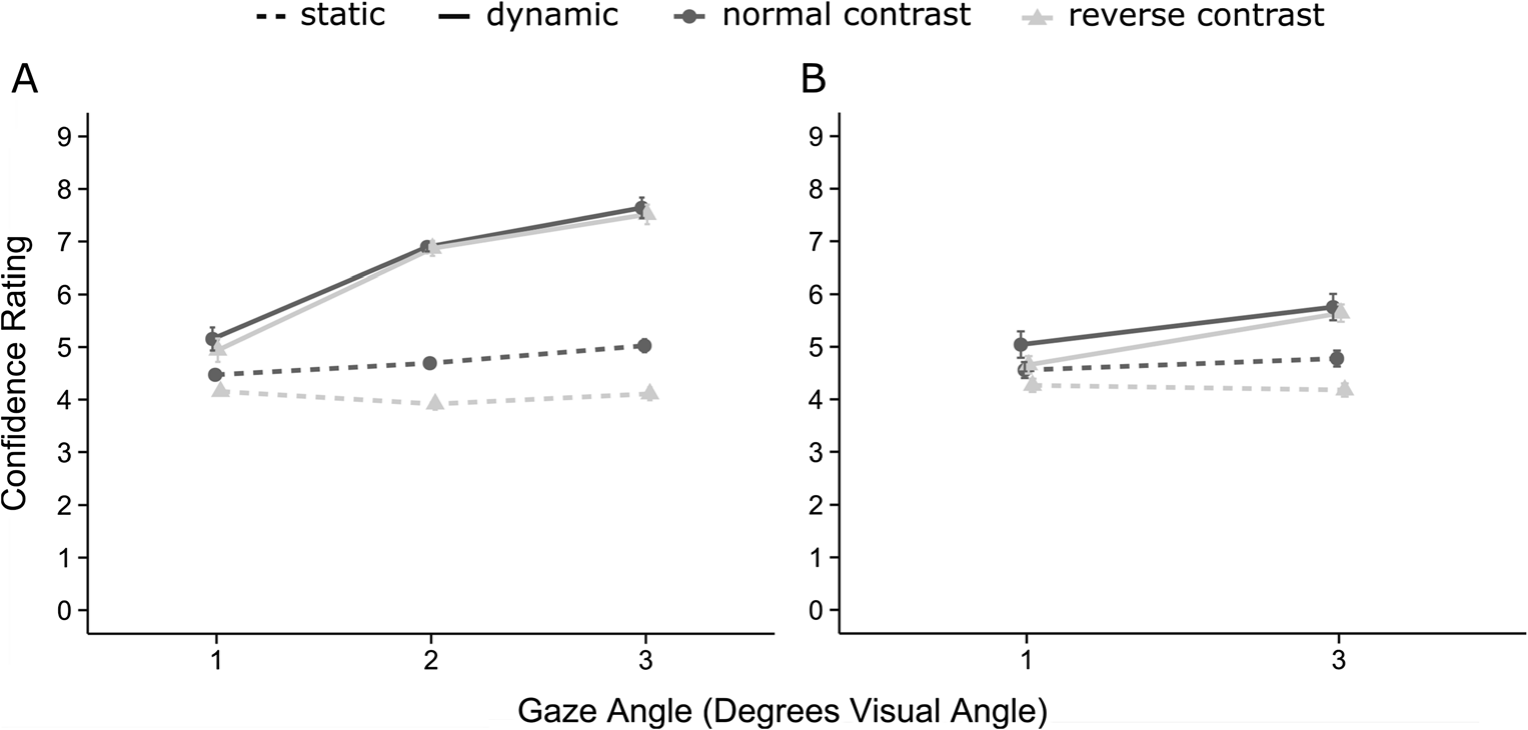
Confidence for normal and reverse contrast gaze in the static and dynamic conditions for each gaze angle for Experiment 1A (**a**) and Experiment 1B (**b**)

Figure 3a shows mean confidence for normal and reverse contrast, static, and dynamic stimuli across the 3 gaze angles. Confidence responses were submitted to a 2 (contrast) × 2 (motion) × 3 (gaze angle) analysis of variance. There was a main effect of contrast, *F*(1,23) = 8.52, *MSE* = 1.32, *p* = 0.008, *η*_*p*_^*2*^=0.270, such that confidence was significantly higher for normal (*M* = 5.66) than for reverse contrast stimuli (*M* = 5.24). There was a main effect of motion, *F*(1,23) = 44.62, *MSE* = 7.17, *p* < 0.001, *η*_*p*_^*2*^ = 0.660, such that confidence was significantly higher for dynamic (*M* = 6.53) than for static (*M* = 4.41) stimuli. There also was a main effect of gaze angle, *F*(1,23) = 78.24, *MSE* = 0.30, *p* < 0.001, *η*_*p*_^*2*^ = 0.773, such that confidence increased linearly with the size of the model’s eye movement.

There was an interaction between motion and gaze angle, *F*(1,23) = 55.11, *MSE* = 1.13, *p* < 0.001, *η*_*p*_^*2*^ = 0.706; this was qualified by a marginal contrast by motion by gaze angle interaction, *F*(1,23) = 3.18, *MSE* = 1.46, *p* = 0.088, *η*_*p*_^*2*^ = 0.122. To investigate this interaction, the linear slopes relating gaze angle to confidence were calculated for each condition. For static stimuli, the effect of gaze angle on confidence was significantly greater for normal contrast (0.28/degree) compared with reverse contrast (−0.02/degree), *t*(23) = 2.91, *p* = 0.008, *d* = 0.65. For dynamic stimuli, no significant difference was found between normal (1.24/degree) and reverse contrast (1.29/degree) stimuli, *t*(23) = 0.28, *p* = 0.780, *d* = 0.06. Thus, as with the accuracy data, the influence of contrast reversal increased with gaze angle for static but not dynamic stimuli (Fig. 3a).

## Results – Experiment 1B

### Correct direction

Figure 2b shows mean direction accuracy (regardless of whether the response to 1° or 3° was correct). To investigate whether directional accuracy replicated the results of Experiment 1A, a 2 (contrast) × 2 (motion) × 2 (gaze angle) within-subjects analysis of variance was conducted on the proportion of correct direction responses. There was a main effect of contrast, *F*(1,11) = 29.40, *MSE* = 0.02, *p* < 0.001, *η*_*p*_^*2*^ = 0.728, such that direction accuracy was higher for normal contrast (*M* = 0.82) than reverse contrast stimuli (*M* = 0.72). There also was a main effect of motion, *F*(1,11) = 112.68, *MSE* = 0.026, *p* < 0.001, *η*_*p*_^*2*^ = 0.911, such that direction accuracy was higher for dynamic (*M =* 0.89) compared with static stimuli (*M* = 0.65). There was a main effect of gaze angle, *F*(1,11) = 36.92, *MSE* = 0.01, *p* < 0.001, *η*_*p*_^*2*^ = 0.770, such that direction accuracy was higher for gaze angles of 3° (“far” gaze; *M* = 0.82) compared with 1° (“near” gaze, *M* = 0.73).

Critically, as in Experiment 1A, there was a significant contrast by motion interaction, *F*(1,11) = 14.87, *MSE* = 0.02, *p* = 0.003, *η*_*p*_^*2*^ = 0.575, such that the effect of the reverse contrast manipulation was larger in the static condition (*M*_*diff*_=0.18) than in the dynamic condition (*M*_*diff*_ = 0.03), *t*(11) = 3.86, *p* = 0.003, *d* = 1.11. Indeed, there was no difference between normal and reverse contrast stimuli in the dynamic condition, *t*(11) = 1.27, *p* = 0.231, *d* = 0.37, whereas this difference was significant in the static condition, *t*(11) = 5.61, *p* < 0.001, *d =* 1.62.

There also was an interaction between contrast, motion, and gaze angle, *F*(1,11) = 6.47, *MSE* = 0.005, *p* = 0.027, *η*_*p*_^*2*^ = 0.370. For static stimuli, the effect of gaze angle (mean at 1° subtracted from the mean at 3°) on accuracy was significantly greater for normal contrast (*M*_*diff*_ = 0.18) compared with reverse contrast (*M*_*diff*_ = −0.02) stimuli, *t*(11) = 2.81, *p* = 0.017, *d* = 0.81. For dynamic stimuli, no significant difference between the effect of gaze angle was found between normal (*M*_*diff*_ = 0.08) and reverse contrast (*M*_*diff*_ = 0.11) stimuli, *t*(11) = 1.13, *p* = 0.289, *d* = 0.33. Thus, the influence of contrast reversal increased with gaze angle for static stimuli but not for dynamic stimuli (Fig. 2b).

### Positional accuracy given correct direction

**Fig. 4:**
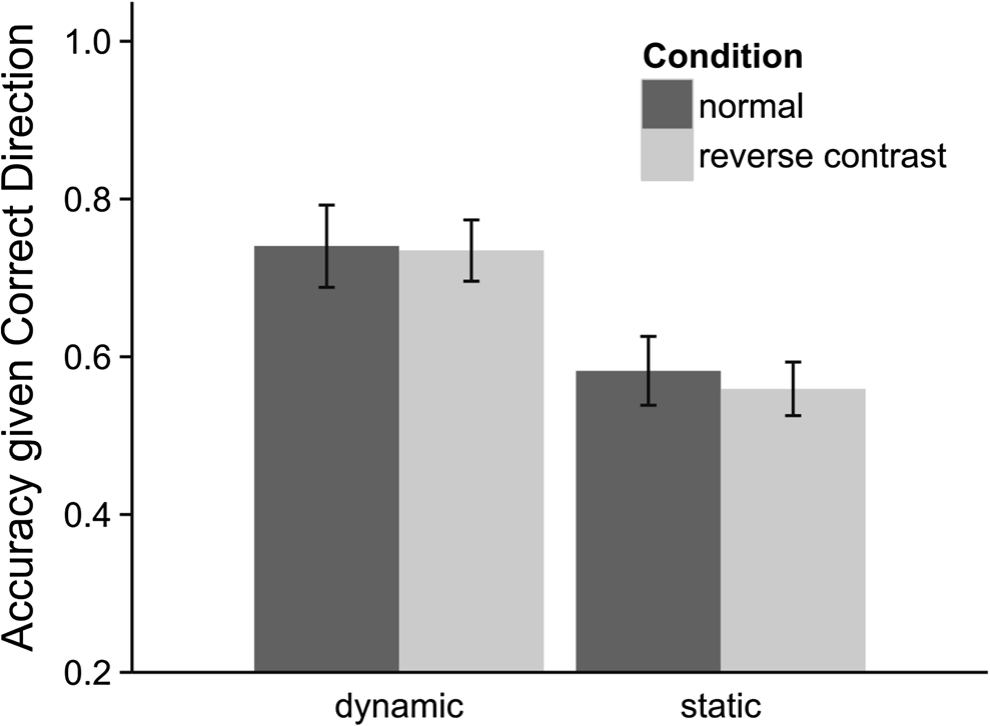
Two-alternative forced-choice discrimination accuracy of gaze position in Experiment 1B, given that participants responded with the correct gaze direction

To more closely investigate the impact of motion and contrast manipulations on participant judgments of gaze location, responses were excluded if participants did not respond with the correct direction. Figure 4 shows the two-alternative forced-choice position discrimination accuracy given that participants made a correct direction judgment across static, dynamic, normal, and reverse contrast stimuli. A 2 (contrast) × 2 (motion) analysis of variance revealed a main effect of motion, *F*(1,11) = 21.69, *MSE* = 0.014, *p* < 0.001, *η*_*p*_^*2*^ = 0.664, such that accuracy was significantly higher for dynamic (*M* = 0.73) compared with static (*M* = 0.57) gaze stimuli. Interestingly, we found no main effect of contrast reversal, *F*(1,11) = 0.224, *MSE* = 0.01, *p* = 0.631, *η*_*p*_^*2*^ = 0.022, nor an interaction between contrast and motion *F* < 1, for gaze position discrimination, which is unlike the significant results for contrast reversal on gaze direction discrimination that we recorded in both Experiments 1A and 1B.

### Confidence

Figure 3b shows mean confidence for normal and reverse contrast, static, and dynamic stimuli. Confidence responses were submitted to a 2 (contrast) × 2 (motion) analysis of variance. There was a main effect of motion, *F*(1,11) = 6.01, *MSE* = 1.37, *p* = 0.032, *η*_*p*_^*2*^ = 0.353, such that confidence was higher for dynamic (*M* = 5.27) compared with static stimuli (*M* = 4.44). There was a marginal effect of contrast reversal, *F*(1,11) = 3.80, *MSE* = 0.38, *p* = 0.077, *η*_*p*_^*2*^ = 0.257, such that confidence was higher for normal (*M* = 5.03) compared with reverse contrast stimuli (*M* = 4.68).

## General discussion

The present investigation has yielded several new findings regarding the influence of motion on gaze discrimination. First and foremost, motion significantly influenced both accuracy and confidence for left/right gaze direction discrimination judgments and gaze position judgments within a visual field (1° vs. 3°). This provides strong evidence that motion can function as a cue in determining gaze direction. One of the most surprising results was the elimination of the effect of contrast reversal on gaze direction discrimination accuracy and confidence when a motion cue was available, a finding that replicated across both Experiments 1A and 1B. There was, however, a large detrimental effect of reverse contrast in the static condition (as others have demonstrated; Ricciardelli et al., 2000; Sinha, 2000). Interestingly, contrast reversal had little impact in Experiment 1B on gaze position discrimination accuracy within a visual field, suggesting the importance of this luminance information for directional, rather than positional gaze discrimination. Taken together, these data confirm that motion is an important cue used in the perception of gaze. In the following, we explore the implications of the present results for developing theories of gaze perception.

### Multiple cues in gaze discrimination

The present findings diverge from a number of studies that found that motion had no effect in tasks that involve precise gaze triangulation (e.g., looking to specific target pegs on a board; Symons et al., 2004; Bock et al., 2008). With the different task used here (judging direction and relative position rather than gaze triangulation), motion improved both accuracy in determining left/right gaze direction and more fine positional information (e.g. looking 1° or 3° within a visual field). Furthermore, at least in the context of simple left/right discrimination, the negative effects of contrast reversal apparent with static stimuli are eliminated when a consistent motion cue is presented. However, regardless of whether gaze was static or dynamic, when judging gaze position, the luminance distribution had little impact on performance (Fig. 4). This suggests that the contribution of the luminance cue may depend on the relative fineness of the gaze judgment to be made. While it is has an impact on the coarser judgments of gaze *direction*, it has little on the finer judgments of gaze *position*. These data fit naturally with the idea that the visual system uses multiple cues in making gaze discriminations depending on the specific task or judgment required (Jenkins, 2007; Olk et al., 2008). In doing so, the present study opens the door to a range of future questions, such as: what is the combined effect of different motion signals to gaze discrimination, as when head motion is in one direction and gaze motion is in another; what is the relationship between these different motion signals and their congruency with the final gaze position and luminance distribution across the eye; and is the contribution of motion to gaze discrimination dependent on the specific type of discrimination that is required?

Future work will need to examine the dynamics of integration across cue types in gaze perception within a multiple-cue framework. For example, though motion cues significantly enhanced the perception of left/right deviations in gaze, the possibility remains that luminance cues are more important than motion cues with larger gaze deviations and/or at longer viewing distances.

### Accuracy versus confidence

The purpose of including confidence responses was to investigate the influence of the various manipulations on individuals’ subjective perception of the gaze “signal.” As might be expected, confidence mirrored accuracy in almost every respect, increasing with the presence of the motion signal, the size of the gaze angle, and when luminance was normal. One interesting exception concerns the dissociation between accuracy and confidence in the reverse contrast condition in Experiment 1A and the correct direction results of Experiment 1B. Here, as accuracy increased with gaze angle, the confidence in those decisions remained low. This dissociation suggests that the perception of gaze direction is not necessarily tied to the conscious “awareness” of where someone is looking. This converges with the notion that gaze direction discrimination is a fundamental perceptual process.

## Conclusions

The results of the present investigation shed new light on the nature of gaze perception. Participants can rely on the motion of the eyes to make both gaze direction and position discrimination judgments. Furthermore, motion can override contradictory luminance cues in the perception of gaze direction and potentially can contribute to judgments outside of the participant’s awareness. Together, these data support the idea that the visual system uses multiple cues to form a coherent perception of gaze direction and that subtle differences in these cues can have a profound effect on how we perceive the eyes of others.

## Footnotes

1 In a preliminary experiment, a static condition was used in which straight and averted gaze frames were separated by a 200 ms blank screen. This equated the static and dynamic stimuli, such that the static condition also included a straight-ahead gaze stimulus. However, performance was disrupted to chance levels in the static condition for both the normal and reverse contrast conditions. Therefore we reverted to the standard methodology for the static condition in the present work.
